# Transports of acetate and haloacetate in *Burkholderia* species MBA4 are operated by distinct systems

**DOI:** 10.1186/1471-2180-12-267

**Published:** 2012-11-20

**Authors:** Xianbin Su, Ka-Fai Kong, Jimmy SH Tsang

**Affiliations:** 1 Molecular Microbiology Laboratory, School of Biological Sciences, The University of Hong Kong, Pokfulam Road, Hong Kong, Hong Kong

## Abstract

**Background:**

Acetate is a commonly used substrate for biosynthesis while monochloroacetate is a structurally similar compound but toxic and inhibits cell metabolism by blocking the citric acid cycle. In *Burkholderia* species MBA4 haloacetate was utilized as a carbon and energy source for growth. The degradation of haloacid was mediated by the production of an inducible dehalogenase. Recent studies have identified the presence of a concomitantly induced haloacetate-uptake activity in MBA4. This uptake activity has also been found to transport acetate. Since acetate transporters are commonly found in bacteria it is likely that haloacetate was transported by such a system in MBA4.

**Results:**

The haloacetate-uptake activity of MBA4 was found to be induced by monochloroacetate (MCA) and monobromoacetate (MBA). While the acetate-uptake activity was also induced by MCA and MBA, other alkanoates: acetate, propionate and 2-monochloropropionate (2MCPA) were also inducers. Competing solute analysis showed that acetate and propionate interrupted the acetate- and MCA- induced acetate-uptake activities. While MCA, MBA, 2MCPA, and butyrate have no effect on acetate uptake they could significantly quenched the MCA-induced MCA-uptake activity. Transmembrane electrochemical potential was shown to be a driving force for both acetate- and MCA- transport systems.

**Conclusions:**

Here we showed that acetate- and MCA- uptake in *Burkholderia* species MBA4 are two transport systems that have different induction patterns and substrate specificities. It is envisaged that the shapes and the three dimensional structures of the solutes determine their recognition or exclusion by the two transport systems.

## Background

*Burkholderia* species MBA4 is a Gram-negative bacterium enriched from soil using monobromoacetate (MBA) as the sole carbon and energy source for growth. MBA4 can also utilize other haloacids such as monochloroacetate (MCA), 2-monochloropropionate (2MCPA) and 2-monobromopropionate (2MBPA) [1]. Since haloacids are environmental pollutants [2-5] and are potentially hazardous for many living organisms [6-8], it is crucial to identify and characterize bacteria that can degrade these alkanoates. The ability for MBA4 to utilize haloacids is conferred by a 2-haloacid dehalogenase Deh4a [1] which has been well characterized [9-11]. A haloacid permease gene, *deh4p*, which forms an operon with *deh4a*, was identified by means of chromosome walking [12]. The function of Deh4p was confirmed by heterologous expression in *E. coli*[13], and its topology determined with a PhoA-LacZ dual reporters system [14]. Further characterization of MBA4 showed that a Deh4p paralog, designated as Dehp2, is also playing a role in MCA uptake. The functional role of Dehp2 was confirmed by gene disruption and heterologous expression in *E. coli*. Single disruptants of *deh4p* or *dehp2* were found to have 30% less of MCA-uptake activity. Moreover, cells with a disrupted *deh4p* gene have an enhanced expression in *dehp2* and vice versa. It looks like Deh4p has a higher affinity for MCA while Dehp2 prefers chloropropionate. When a *deh4p*^‒ ^*dehp2*^‒ ^double disruptant was constructed, the cells still retain 36% of MCA-uptake activity. It was concluded that a robust system is present for haloacid uptake in MBA4 [15].

In the process of characterizing the MCA-uptake activity of MBA4, it was found that acetate was also recognized by the MCA-inducible uptake system [12,15]. Since acetate and MCA are structurally similar, it is reasonable to speculate that MCA was transported by an acetate-transport system. It has been reported that acetate could freely diffuse across the cell membrane in an un-disassociated form (acetic acid) [16]. However, in growth conditions with a neutral pH where acetate is mainly in a disassociated form, a specific transport system is needed. There are reports leading to the identification of acetate permeases in many bacterial species, including ActP in Gram-negative *E. coli *[17] and MctC in Gram-positive *Corynebacterium glutamicum *[18]. As MBA4 can grow on acetate, it is likely that an acetate-transport system is also present. Whether this acetate-transport system is playing a role in MCA uptake is important to the understanding of the MCA-uptake system in MBA4.

In this study, we analyzed the induction patterns of the acetate- and MCA-uptake systems and determined the substrate specificities of the two systems in cells grown in various substrates. We demonstrated that there are distinct acetate- and MCA- transport system in MBA4. Nonetheless, both systems were sensitive to carbonyl cyanide *m*-chlorophenyl hydrazone indicating that transmembrane electrochemical potential is a driving force for both systems.

## Results

### The induction patterns of acetate- and MCA- transport systems are different

**Figure 1:**
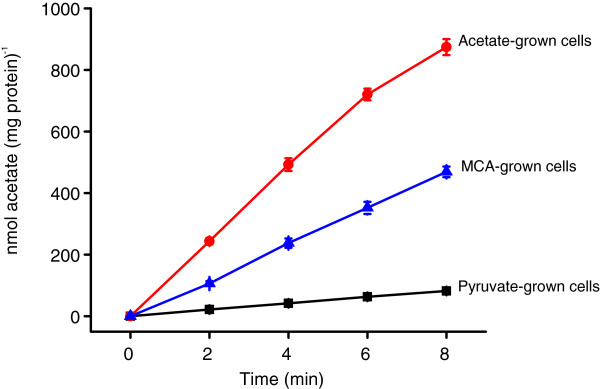
**Acetate-uptake activity of MBA4. MBA4 was grown in minimal medium containing pyruvate (squares), acetate (circles), or MCA (triangles).** Uptake of 50 μM of [2-^14^C]acetate was assayed by a filtration method for a period of 8 min. Data shown are the means of three independent experiments, and the error bars represent the standard deviations.

Previous analysis showed that MCA-uptake activity of MBA4 was detected only in MCA- and not in acetate-grown cells [12]. This indicated that the MCA-uptake activity was induced by MCA and not by acetate. It was also shown that MCA-grown cells possess acetate-uptake activity [12]. To check whether a distinct acetate-transport system is present in MBA4, acetate-uptake assays were carried out for pyruvate-, acetate-, and MCA-grown cells. The results showed that pyruvate-grown cells had no detectable activity, while both acetate- and MCA-grown cells had significant acetate-uptake activities (Figure 1). Acetate-grown cells had an acetate-uptake rate of 111.27 nmol (mg protein)^-1^ min^-1^ for the first 8 min, and MCA-grown cells had a rate of 59.20 nmol (mg protein)^-1^ min^-1^. This indicated that acetate was not entering the cells passively, and there is an inducible acetate-transport system in MBA4.

**Figure 2:**
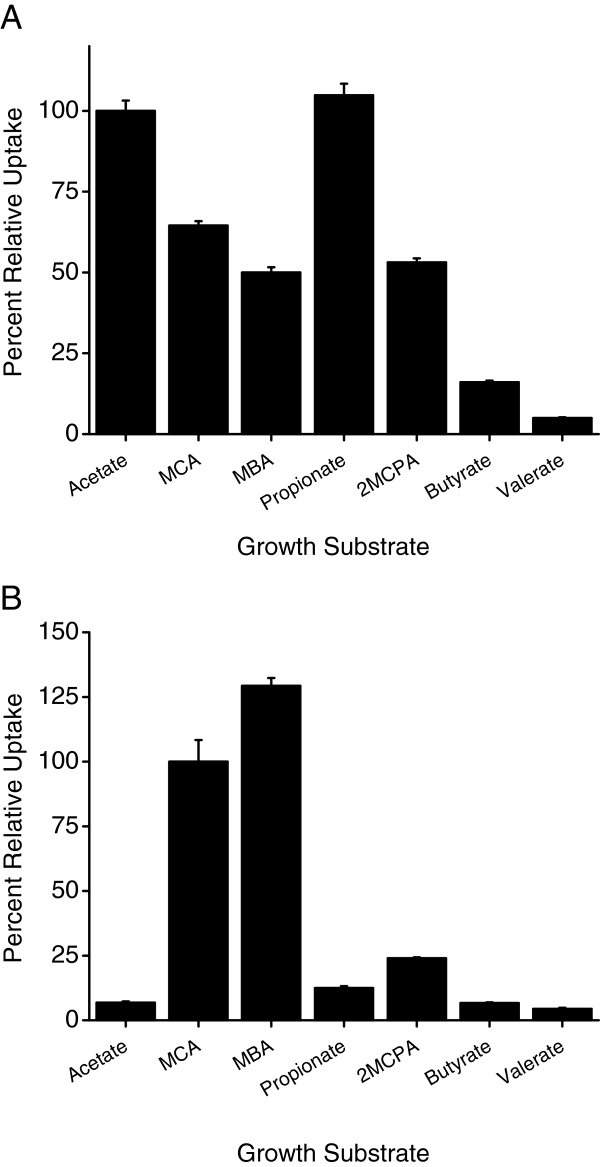
**Relative acetate- and MCA- uptake activities of MBA4 grown in various substrates.** MBA4 was grown in minimal medium containing acetate, MCA, MBA, propionate, 2MCPA, butyrate, or valerate. Uptakes of 50 μM of [2-^14^C]acetate (**A**) or [2-^14^C]MCA (**B**) were assayed for a period of 1 min. Data shown are the means of three independent experiments, and the error bars represent the standard deviations. (**A**) The acetate-uptake rate of acetate-grown cells was set as 100%, and acetate-uptake rates of cells grown in other substrates were determined and shown as percentages. (**B**) The MCA-uptake rate of MCA-grown cells was set as 100%, and MCA-uptake rates of cells grown in other substrates were determined and shown as percentages.

In order to characterize the inducible acetate-uptake system, MBA4 cells were grown in various carbon sources and their relative acetate-uptake activities, using acetate-grown cells as the standard, determined. Figure 2A shows that propionate induced similar level of uptake activity while MCA, MBA and 2MCPA only induced around 50% of the standard. Butyrate and valerate induced less than 20% of activity. As a comparison, cells were also grown in similar substrates and their relative MCA-uptake activities determined. Figure 2B shows that MBA induced comparable MCA-uptake activity as MCA but 2MCPA only induced about 20% of activity. The inductions conferred by acetate, propionate, butyrate, and valerate were rather minimal and only represent a mere 10% or less. As the MCA-uptake activity was induced significantly only by monohaloacetate while the acetate-uptake activity induced by acetate, haloacetate, propionate and 2MCPA, the induction patterns of the two transport systems appear to be different.

### Acetate- and MCA- transport systems have different substrate specificities

In order to conclude that the transports of acetate and MCA were executed by different systems, competing solute analysis was used to deduce the substrate specificities of the induced acetate- and the MCA- transport systems in MBA4. Acetate uptakes were determined for both acetate- and MCA-grown cells. MCA uptakes were determined only for MCA-grown cells because acetate-grown cells have no MCA-uptake activity. Competing solutes that exhibit structural similarity to acetate or propionate were selected.

**Figure 3:**
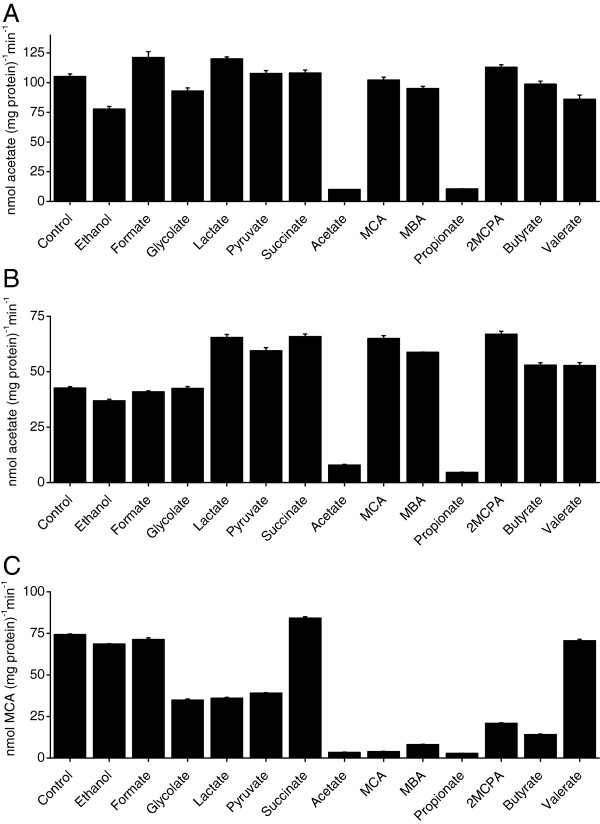
**Inhibition of acetate- and MCA- uptake by other solutes. Uptakes of 50 μM of [2-**^**14**^**C] labelled acetate or MCA were determined in the presence of competing solutes.** The assays were conducted for 1 min. Competing solutes were added to a final concentration of 0.5 mM. Competing solutes used were: ethanol, formate, glycolate, lactate, pyruvate, succinate, acetate, MCA, MBA, propionate, 2MCPA, butyrate, and valerate. Uptake rates without competitor were used as the controls. Data shown are the means of three independent experiments, and the error bars represent the standard deviations. (**A**) Acetate uptake of acetate-grown cells; (**B**) Acetate uptake of MCA-grown cells; (**C**) MCA uptake of MCA-grown cells.

Acetate uptake of acetate-grown cells was significantly inhibited by acetate and propionate, with an inhibition of 91% and 90%, respectively (Figure 3A). When MCA-grown cells were used, a similar pattern was observed for acetate uptake. Only acetate and propionate served as effective inhibitors (Figure 3B). When MCA-grown cells were used for MCA-uptake assays, acetate, MCA, MBA, propionate, 2MCPA and butyrate acted as efficient inhibitors. In addition, glycolate, lactate, and pyruvate also had moderate inhibitory effects on MCA uptake as previously reported [12] (Figure 3C). These results showed that the acetate-uptake activity was inhibited only by acetate and propionate while the MCA-transport system was inhibited by substrates that display a similar structure as haloacetate.

### Transmembrane electrochemical potential is a driving force for both acetate- and MCA- transport

**Figure 4:**
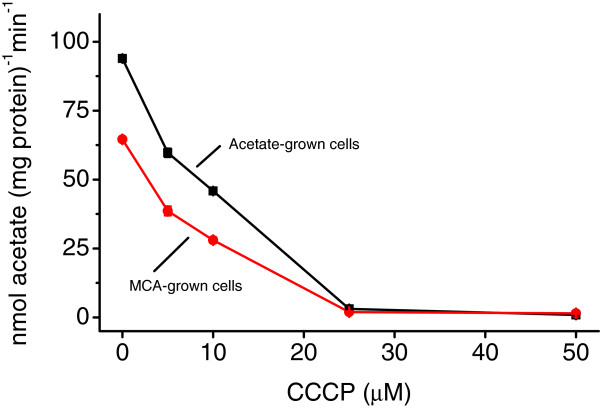
**Effect of CCCP on acetate uptake. MBA4 was grown in minimal medium containing acetate (squares) or MCA (circles).** Uptakes of 50 μM of [2-^14^C]acetate were assayed in the presence of 0, 5, 10, 25, and 50 μM of CCCP for a period of 1 min. Data shown are the means of three independent experiments, and the error bars represent the standard deviations.

During the characterization of the haloacid operon of MBA4, a protonophore, carbonyl cyanide *m*-chlorophenyl hydrazone (CCCP), was shown to abolish the MCA-uptake activity of MBA4 (M. Yu, unpublished). The effect of CCCP on acetate uptake was duly investigated. Figure 4 shows that the inclusion of increasing amount of CCCP in uptake assays for acetate- and MCA-grown cells, the acetate-uptake rates decreased accordingly. The uptake activities were completely abolished when 25 μM of CCCP were supplemented in the reactions. As CCCP collapses the proton gradients across the cell membrane [19], acetate uptake in MBA4 is likely to be dependent on the transmembrane electrochemical potentials, a condition similar to that of MCA uptake.

**Figure 5:**
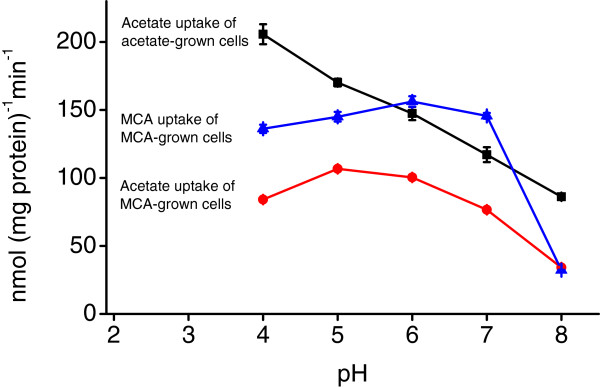
**Effect of pH on acetate- and MCA- uptake.** MBA4 was grown in minimal medium containing acetate or MCA, harvested and resuspended in potassium phosphate buffers of various pH values. Uptakes of 50 μM of [2-^14^C] labelled acetate or MCA were assayed for a period of 1 min. Squares represent acetate uptake of acetate-grown cells, circles represent acetate uptake of MCA-grown cells, and triangles represent MCA uptake of MCA-grown cells. Data shown are the means of three independent experiments, and the error bars represent the standard deviations.

A limitation of employing CCCP is that it cannot discriminate between proton-coupled symport and Na^+^-coupled symport [17,20]. As it is difficult to remove sodium from the buffers completely and radioactive MCA and acetate were provided in the form of a sodium salt, the effect of pH on acetate- and MCA- uptake was examined with an aim to find out the possible involvement of proton(s). In acetate uptake of acetate-grown cells, the uptake rate decreased steadily as pH increased from 4 to 8 (Figure 5, squares). In acetate uptake of MCA-grown cells, the uptake rate increased slightly as pH increased from 4 to 5 and then dropped gradually as pH increases (Figure 5, circles). The uptake rates were much lower than that of acetate-grown cells in similar assay conditions. In MCA uptake of MCA-grown cells, the uptake rate increased slightly as pH increased from 4 to 6 and dropped swiftly from pH 7 to 8 (Figure 5, triangles). These results showed that acetate- and MCA- transport systems have different sensitivities to pH. Nonetheless, the involvement of proton(s) in acetate transport is noticeable.

## Discussion

In this study, we demonstrated the presence of distinct acetate- and MCA- transport system in MBA4. This is supported by: (i) the observation that the inducible substrates for acetate- and MCA- uptake activity were different; (ii) the two transport systems have different competing solutes and (iii) a difference in dependency on pH for the two systems. The failure of pyruvate-grown cells to take up acetate suggested that the acetate-transport system in MBA4 was inducible. Both acetate and MCA were able to induce acetate-uptake activity although to a different level. Acetate permease MctC of *Corynebacterium glutamicum* is also inducible. MctC exhibits a high affinity for acetate and propionate and low affinity for pyruvate. In this case, the expression was higher in pyruvate- than in acetate-grown cells. As a result, both pyruvate- and acetate-grown cells showed comparable acetate-uptake activities [18]. In MBA4, no induction was observed for pyruvate while acetate and propionate were the best inducers for acetate uptake. Moreover, they were also the most favourable substrates. It is possible that acetate and propionate were transported by the same transport system but further confirmation is required as *Candidatus* Competibacter phosphatis appeared to have different transporters for the two solutes [21]. Another acetate permease, ActP of *Rhodobacter capsulatus*, was produced around 1.5 folds more in acetate- than in pyruvate-grown cells [22]. This indicated that the regulations of various acetate-transport systems in different bacteria are likely to be different and should be compared cautiously.

**Figure 6:**
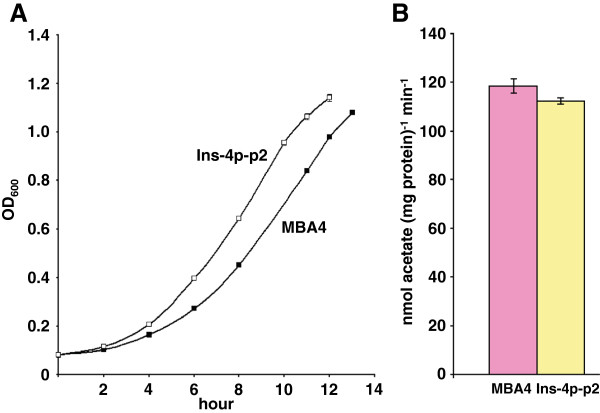
**Growth on and uptake of acetate of a *deh4p***^**– **^***dehp2***^**– **^**double mutant.** (**A**) Wildtype MBA4 (■) and *deh4p*^– ^*dehp2*^– ^double mutant (□) were grown in minimal medium containing acetate. Seed cultures were grown in LB^– ^and sub-cultured into minimal medium containing acetate at 30°C. The optical densities of the cultures were determined at 600 nm (OD_600_) with a spectrophotometer. (**B**) Acetate-uptake rates of acetate-grown- wildtype and double mutant. Uptakes of 50 μM of [2-^14^C]acetate were assayed by a filtration method for a period of 2 min. Data shown are the means of three independent experiments, and the error bars represent the standard deviations.

It is not surprising that MCA-grown cells could take up MCA and acetate because most transporters recognize more than one substrate. Acetate permease ActP of *E. coli* was able to transport acetate and glycolate [17]. Moreover, acetate and MCA are structurally similar molecules. The ability for MCA-grown cells to transport acetate can be explained by (1) the capability of the induced MCA-transport system to transport acetate; (2) the acetate-transport system was also induced by MCA; and (3) both (1) and (2). Without the identification of the individual permease involved in each of the transport system it is difficult to determine conclusively which the case is. The cloning and heterologous expression of Deh4p in *E. coli* demonstrated its function as a dehalogenase-associated MCA-transporter [13]. Similarly, the functional role of Dehp2 as a second MCA-transporter was also demonstrated [15]. Both Deh4p and Dehp2 were capable of recognizing acetate as a substrate. In order to elucidate that the MCA-uptake system, comprising Deh4p and Dehp2, is not the main transporter for acetate, a *deh4p*^‒ ^*dehp2*^‒ ^double mutant (strain Ins-4p-p2, [15]) was utilized. Figure 6A shows that the growth of Ins-4p-p2 on acetate was similar to that of wildtype MBA4, if not slightly better. The acetate-uptake rate of this acetate-grown mutant was also assayed and shown to be similar to that of wildtype (112.3 nmol (mg protein)^-1^ min^-1^ for mutant and 118.6 nmol (mg protein)^-1^ min^-1^ for wildtype, Figure 6B). This suggested that in the absence of the major players in MCA uptake the growth and uptake activity on acetate of the cell were not affected. This confirmed the presence of an independent acetate-transport system other than the MCA-uptake system.

The competing solute analyses show that acetate- and MCA-grown cells have similar inhibition pattern for acetate uptake. This suggested that the acetate-transport system was likely to be induced by MCA. The relatively lower acetate-uptake rate for MCA-grown cells suggested that MCA was a weaker inducer. This is consistent with the observation that acetate and propionate were the best inducers for acetate uptake. The competing solute analyses for MCA-grown cells show that the cells have different inhibition patterns for acetate- and MCA- uptake. The failure of MCA to inhibit the uptake of acetate suggested that the acetate-transport system was expressed and not involved in MCA transport. This is in agreement with the result that acetate-grown cells failed to transport MCA. The ability for acetate to inhibit the MCA-uptake activity of MCA-grown cells concluded that the MCA-uptake activity is different from the acetate-uptake system.

The effect of pH on the uptakes of acetate of acetate- and MCA-grown cells further demonstrates the presence of two systems. The uptake rates of acetate-grown cells decrease linearly with an increase in pH. This shows that proton plays an essential role in the acetate-uptake system. In this condition no MCA-uptake system was produced. When the cells were grown on MCA the rates of acetate uptake on different pH deviate from that of acetate-grown cells. The competing solute analysis demonstrated a similar pattern of inhibition on acetate uptake for acetate- and MCA-grown cells while the rate was much lower for the latter. It is most likely that the expression of the acetate-uptake system was lower in MCA-grown cells. In this case, the major transport system was that for MCA and which can also transport acetate. Since both acetate- and MCA- transport systems are proton dependent, the pH dependency of acetate uptake of MCA-grown cells was thus exhibiting a pattern different from that of acetate-grown cells and was displaying a hybrid pattern between acetate uptake of acetate-grown cells and MCA uptake of MCA-grown cells. Future experiments that assay the pH dependency of acetate uptake of MCA-grown Ins-4p-p2 double mutant could clarify the situation. However, the expressions of other transporters may be affected by the disruptions of *deh4p* and *dehp2 *[15] and could complicate the outcome. Moreover, when the gene responsible for the acetate-uptake system has been identified, it is necessary to measure its expression levels in medium containing acetate, MCA and other substrates in order to characterize the system fully.

**Figure 7:**
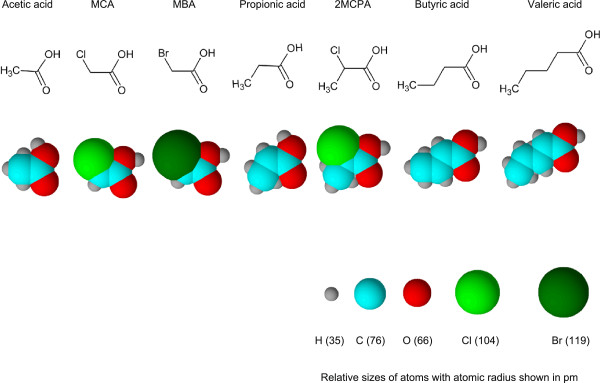
**Structural models of various competing solutes.** The values of atomic radii, the skeletal formula and the space-filling models of acetic acid, MCA, MBA, propionic acid, 2MCPA, butyric acid, and valeric acid were obtained from ACD/ChemSketch (Advanced Chemistry Development, Inc.). The solutes were assumed to be in disassociated forms in PBS buffer (pH 7.4) used in this study.

The most distinct difference between the two transport systems is their substrate specificity. The failure of ethanol to inhibit acetate transport suggested that the carboxyl group is likely to be an important element. The lack of inhibition by formate implied that the presence of a second carbon is also essential. The failure of MCA, MBA, and 2MCPA to inhibit acetate transport indicated that the substituted halogen atom affected the effective recognition. The acetate-uptake ability of MBA4 was inhibited by propionate but not by butyrate. This is consistent with the acetate permease ActP of *E. coli *[17]. The failure of butyrate and valerate to act as a competing solute suggested that a molecule with more than three carbons would be less effective for the acetate-uptake system. In summary, MCA, MBA, 2MCPA, and butyrate could inhibit MCA- but not acetate- uptake of MBA4. A visual inspection of the structural models of these molecules (Figure 7) suggests that they are generally larger than acetate. Similarly, MCA, MBA, and propionate have a stronger inhibitory effect on MCA uptake than 2MCPA and butyrate. The failure of valerate to act as a competing solute further strengthens the notion that size is a determining factor. By means of comparing the structures of the competing solutes it may be possible to estimate the range of substrates recognized by various transport systems and provide valuable information on the functional property of the transporters.

The inducers for the acetate-uptake system are acetate, MCA, MBA, propionate, and 2MCPA, but only acetate and propionate are substrates. Similarly, the inducers for the MCA uptake system are MCA, MBA and to a lesser extend 2MCPA, while the substrates include the inducers, acetate and propionate. The inducer and the substrate are not necessarily the same. Although the acetate- and the MCA- transport systems have different induction patterns and substrate specificities, they do share certain similarity. The activities of both systems were abolished by CCCP, suggesting transmembrane electrochemical potential as a driving force. As CCCP could not discriminate between proton- and sodium-coupled symport, it was unclear which was/were involved in the transports. Previous studies of bacterial acetate-transport systems failed to give a uniformed conclusion. Although ActP of *E. coli* was assigned to the sodium:solute symporter family, no dependency on sodium was demonstrated [17]. While electrochemical proton potential was confirmed to be a driving force for MctC of *Corynebacterium glutamicum *[18], acetate uptake in *Accumulibacter* spp. was believed to be driven by proton motive force, and in *Defluviicoccus* spp. it was suggested to be powered by proton or sodium gradient or both [23]. An increased uptake of acetate for a change of pH from 8 to 4 affirmed the involvement of proton in acetate transport in MBA4. However, the involvement of sodium could not be ruled out and further confirmation is required.

## Conclusions

The uptakes of acetate and MCA in *Burkholderia* species MBA4 were demonstrated to be manoeuvred by different transport systems. These systems have different induction patterns and substrate specificities. A driving force for both systems is transmembrane electrochemical potential, and proton is involved in acetate transport. A structural comparison of the competing solutes suggests that the size of the molecule is a determinant factor for recognition. Future work on identification and characterization of the transporter protein is required to understand the systems comprehensively.

## Methods

### Bacterial strains and culture conditions

*Burkholderia* species MBA4 and mutant Ins-4p-p2 were grown at 30°C in Luria Bertani medium without NaCl (LB^–^, 1% tryptone, 0.5% yeast extract) or in defined minimal medium [1] with 0.5 g carbon liter^-1 ^of pyruvate, acetate, MCA, MBA, propionate, 2MCPA, butyrate, or valerate.

### Transport assays

MBA4 was cultured in minimal medium with pyruvate, acetate, MCA, MBA, propionate, 2MCPA, butyrate, or valerate to late logarithmic phase, with an optical density value (OD_600_) of 1.0-1.2, 0.9-1.1, 0.5-0.7, 0.7-0.9, 0.9-1.1, 0.1-0.2, 0.9-1.1 or 0.9-1.1, respectively. Cells were harvested by centrifugation, washed twice with phosphate buffered saline (PBS, Fluka), and adjusted to an OD_600_ of around 0.4. For standard transport assays, 30 μl of [2-^14^C]MCA (Sigma-Aldrich, diluted to 0.25 mM in PBS) or [2-^14^C]acetate (Sigma-Aldrich, diluted to 0.25 mM in PBS) were added to 120 μl of prepared cells, mixed, and 30 μl samples were taken at various time points. Filtration and washing of cells, determinations of total protein and trapped [2-^14^C]MCA or [2-^14^C]acetate were carried out as previously described [12].

To determine the substrate specificity, diluted [2-^14^C]MCA or [2-^14^C]acetate was mixed with 10× competing solutes in PBS before adding to the prepared cells. Percent relative uptake was calculated as (Uptake rate with competing solute/Uptake rate without competing solute) × 100%. The competing solutes included: ethanol; one-carbon monocarboxylate formate; two-carbon glycolate, acetate, MCA and MBA; three-carbon propionate, lactate, pyruvate and 2MCPA; four-carbon butyrate, five-carbon valerate; and four-carbon dicarboxylate succinate. The skeletal formulas and space-filling models of acetic acid, MCA, MBA, propionic acid, 2MCPA, butyric acid, and valeric acid were drawn with ACD/ChemSketch (Advanced Chemistry Development, Inc.).

To study the effect of protonophore on uptake assay, appropriate amounts of carbonyl cyanide *m*-chlorophenyl hydrazone (CCCP) were mixed with prepared cells to a final concentration of 0, 5, 10, 25, and 50 μM for 30 min before transport assays were conducted.

To determine the effect of pH on transport systems, 100 mM potassium phosphate buffers of different pH values (4 to 8) were used to resuspend the bacterial cells and for diluting [2-^14^C]MCA and [2-^14^C]acetate for uptake assays.

## Abbreviations

MCA: Monochloroacetate; MBA: Monobromoacetate; 2MCPA: 2-Monochloropropionate; 2MBPA: 2-Monobromopropionate; CCCP: Carbonyl Cyanide *M*-Chlorophenyl Hydrazone; PBS: Phosphate Buffered Saline.

## Competing interests

The authors declare that they have no competing interests.

## Authors contributions

XS and KFK designed and carried out the studies and drafted the manuscript. JSHT conceived of the study, participated in the design and coordination of the study and drafted the manuscript. All authors read and approved the final manuscript.
